# Personality and weight management in adults with type 2 diabetes: A systematic review

**DOI:** 10.3389/fcdhc.2022.1044005

**Published:** 2022-11-11

**Authors:** Ralph Geerling, Emily J. Kothe, Jeromy Anglim, Catherine Emerson, Elizabeth Holmes-Truscott, Jane Speight

**Affiliations:** ^1^ School of Psychology, Deakin University, Geelong, VIC, Australia; ^2^ The Australian Centre for Behavioural Research in Diabetes, Diabetes Victoria, Melbourne, VIC, Australia

**Keywords:** type 2 diabetes, personality, obesity, overweight, weight management, health behaviours

## Abstract

**Aims:**

Managing weight in the context of type 2 diabetes presents unique hormonal, medicinal, behavioural and psychological challenges. The relationship between weight management and personality has previously been reviewed for general and cardiovascular disease populations but is less well understood in diabetes. This systematic review investigated the relationship between personality constructs and weight management outcomes and behaviours among adults with type 2 diabetes.

**Methods:**

Medline, PubMed, Embase, PsycINFO and SPORTDiscus databases were searched to July 2021. Eligibility: empirical quantitative studies; English language; adults with type 2 diabetes; investigation of personality-weight management association. Search terms included variants of: diabetes, physical activity, diet, body mass index (BMI), adiposity, personality constructs and validated scales. A narrative synthesis, with quality assessment, was conducted.

**Results:**

Seventeen studies were identified: nine cross-sectional, six cohort and two randomised controlled trials (N=6,672 participants, range: 30-1,553). Three studies had a low risk of bias. Personality measurement varied. The Big Five and Type D personality constructs were the most common measures. Higher emotional instability (neuroticism, negative affect, anxiety, unmitigated communion and external locus of control) was negatively associated with healthy diet and physical activity, and positively associated with BMI. Conscientiousness had positive associations with healthy diet and physical activity and negative associations with BMI and anthropometric indices.

**Conclusions:**

Among adults with type 2 diabetes, evidence exists of a relationship between weight management and personality, specifically, negative emotionality and conscientiousness. Consideration of personality may be important for optimising weight management and further research is warranted.

**Systematic review registration:**

www.crd.york.ac.uk/prospero/, identifier CRD42019111002.

## 1 Introduction

Approximately 90% of people with type 2 diabetes are living with obesity or are overweight (1), and less than 30% meet physical activity and dietary recommendations (2, 3). Maintaining physical activity, a healthy diet and a healthy body weight are key recommendations for the optimal management of type 2 diabetes (4), with Australian and international guidelines recommending 3-15% weight loss for people with type 2 diabetes living with obesity or who are overweight (5). A vast body of evidence demonstrates multifaceted barriers to the adoption and maintenance of weight management behaviours (6). For people with type 2 diabetes, unique weight management challenges exist, e.g. prescription medications with weight-gain inducing side-effects (7). These challenges can have compounding behavioural and psychological sequelae including reduced motivation and depression, creating a negative cycle (7, 8). For example, insulin is associated with a mean ± SD weight gain of 4.3 ± 2.7kg overall, and up to 14.7kg in the first year (9). Excess weight or weight gain can lead to cardiovascular disease, depression and reduced quality of life (6). Conversely, reduced engagement in weight management behaviours may be a consequence of impaired emotional wellbeing, including diabetes distress (10), and other psychological factors such as self-efficacy (11), and personality (12).

Personality refers to an individual’s characteristic set of behaviours, cognitions, and emotional patterns that evolve from biological and environmental factors (13). It is a key determinant of wellbeing in the general population (14). The most widely examined conceptualisation of personality in relation to weight management behaviours and outcomes is the Big Five (15). The Big Five represents a person’s tendencies on five broad and continuous traits: Neuroticism (e.g. anxious, stressed), extraversion (e.g. sociable, active), openness to experience (e.g. open-minded, intellectual), conscientiousness (e.g. disciplined, orderly) and agreeableness (e.g. trusting, caring). To date, the Big Five traits of neuroticism, extraversion and conscientiousness have been most consistently associated with weight management behaviours and outcomes among the general population (12). Specifically health-enhancing behaviours have been associated with higher levels of conscientiousness and health-compromising behaviours have been associated with higher levels of neuroticism and extraversion among populations living with obesity (12).

Some of the earliest research on the relationships between personality and health introduced the concept of locus of control. Locus of control postulates that a person’s perception of events are contingent on either their behaviour and characteristics (internal) or by luck, chance, fate or powerful others (external) (16). A greater internal locus of control has been found to be positively associated with performing health behaviours (17). Conceptually similar, agency and communion, which describe how individuals relate to their social world (18), are also rooted in foundational personality philosophy (19). Measuring unmitigated communion, which describes behaviours that prioritise the care of others to the detriment of the self (20), has been shown to have a negative influence on health. Other early research focused on cardiovascular disease in which Freidman and Rosenman introduced the Type A/Type B model of personality (21). Type A personality is characterised as being competitive, ambitious and acting with a sense of urgency, while type B personality is characterised as being more relaxed, less hurried and exhibiting less hostility; with type A being linked to cardiovascular disease (22).

This typological view of the relationship between cardiovascular disease and personality has continued through the development of the distressed personality type, or Type D personality. Type D personality is characterised as an interplay between negative affect, the tendency to experience negative mood and emotions, and social inhibition, a tendency to inhibit self-expression in social situations (23). People with cardiovascular disease who score high for Type D personality, especially negative affect, report sub-optimal physical activity and diet (24).

Given the weight management challenges unique to diabetes, it is unclear whether the personality-weight management relationships observed among the general or cardiovascular disease populations are relevant to the type 2 diabetes population. Among people with type 2 diabetes, there is evidence of an association between low conscientiousness, high neuroticism, or the presence of Type D personality, and increased risk of sub-optimal medication taking, HbA1c, blood glucose monitoring, and complication screening (25–27). Meeting the frequent daily, and challenging, demands of diabetes is burdensome. Certain personality traits have been shown to relate to resiliency (28) and coping strategies (29). Given the psychological and physiological complexities of weight management, deepening our understanding of the relationship between weight management and personality may inform more effective self-management interventions. However, despite the unique challenges and the clinical significance of weight management behaviours and outcomes in type 2 diabetes, comparatively few studies have examined the relationship between personality and weight management specifically and this research has not yet been synthesised.

The aim of this systematic review is to summarise and critically examine the evidence regarding the relationship between personality and weight management behaviours and outcomes in adults with type 2 diabetes.

## 2 Materials and methods

The reporting of this systematic review was guided by the Preferred Reporting Items for Systematic Reviews and Meta-Analyses (PRISMA) statement (30) (see **Supplementary Material 1** for PRISMA checklist). The review protocol was registered on the International Prospective Register of Systematic Reviews (PROSPERO ID: CRD42019111002 www.crd.york.ac.uk/prospero/).

### 2.1 Search strategy

A systematic search was conducted in July 2019 (updated July 2021) to identify peer-reviewed, empirical studies, published in English, that have examined the relationship between personality and weight management in adults (aged 18+ years) with type 2 diabetes. MEDLINE Complete, CINAHL complete, PsycINFO, Embase and SPORTDiscus were searched (since database inception) using terms relating to two themes (1): Personality; and (2) type 2 diabetes. Terms within each theme were combined using the Boolean operator ‘OR’, and the two themes were then combined using the ‘AND’ operator. A full search strategy is provided in **Supplementary Material 2**.

### 2.2 Selection criteria

The population, intervention (or exposure), comparison, outcome (PICO) model was used to guide the search. Refer to **Supplementary Material 2** for full details of the search strategy and terms. Studies were eligible if they:

1. reported results for adults with type 2 diabetes (population);
2. measured personality using a validated personality assessment (e.g. NEO PI-R or DS14), including individual traits, aspects pertaining to temperament and disposition (e.g. anxiousness), and concepts grounded in personality literature (e.g. locus of control) (intervention [or exposure] and comparison where applicable for study design, e.g. personality tailored intervention vs normal care, Type D personality vs non-Type D personality);
3. quantitatively examined the relationship between personality and weight management outcomes or behaviours (outcome); and
4. were published in a peer-reviewed journal article.

Studies were excluded if they:

1. focused solely on people with other types of diabetes, or did not report the results for adults with type 2 diabetes separately;
2. were not published in English;
3. did not specifically address, or provide analysis of, the relationship between personality and weight management;
4. had a qualitative study design;
5. included individuals aged <18 years without reporting the results for individuals aged 18+ years separately.

Weight management was defined broadly to include physical body weight indicators as well as performance of weight-related behaviours prescribed by relevant government guidelines for physical activity and dietary intake. Assessments may include: physical weight indicators that include self-reported or clinically reported kilograms/pounds, Body Mass Index (BMI), waist circumference, hip circumference, waist-to-hip ratio etc. Weight-related behaviours may include: self-reported physical activity (i.e. assessed by validated questionnaire, e.g. International Physical Activity Questionnaire; IPAQ) or objectively measured physical activity (e.g. activity tracker, step counter); self-reported dietary habits (i.e. assessed by validated questionnaire, e.g. food frequency questionnaire; FFQ) or intake (food diary, study-controlled diet).

### 2.3 Screening

Titles and abstracts were screened independently by the first author and one other author. Full-text article review was conducted by the first and fourth author. Conflicts were resolved through discussion and, where required, in consultation with a third author.

### 2.4 Data extraction and synthesis

All data were extracted manually by the first author, with 50% of studies double-extracted by the fourth author, using a purpose-built template. Conflicts were resolved through discussion and, where required, in consultation with a third author.

Extracted data included reference details, country of origin, study design and method, analyses performed, sample size, participant demographics (e.g. age, gender and education level) and clinical characteristics (e.g. diabetes duration, management strategies and BMI), as well as outcome assessment tools (e.g. self-report questionnaire such as IPAQ, objective measurement such as an electronic activity tracker). Data was extracted regardless of the format reported for each study (e.g. age or BMI presented categorically or continuously). Where data not essential to the review topic (e.g. education) was uncollected or not reported, this was noted in the tabulated output describing the studies (refer to **Tables 1** and **2**).

**Table 1:** Participants’ clinical and demographic characteristics, by study design.

Author, Year, Country, Study design	Sample size Age Gender	Body Mass Index (kg/m^2^)	Diabetes duration and diabetes management	Education level
40, Albargawi et al., 2016, Saudi Arabia Cross-sectional	n= 30 ≤49 years 52% ≥50 years 48% 40% female	Not reported	<1 year, 13% ≥1 year, 87% Management strategies not reported	No school: 13% Primary school: 27% Secondary school: 23% University: 37%
48, Elran-Barak et al., 2019, Israel Cross-sectional	n= 368 Mean 72 ± 4.3 years 42% female	Mean: 28.5 ± 4.3	0-4.99 years: 12.2% 5-9.99 years: 43.9% 10+ years: 43.4% Management strategies not reported	Means years of education 13.5 ± 3.5
41, Köbling et al., 2020 Hungary Cross-sectional	n= 178 Mean 59.2 ± 13.6 years 56.8% female	Mean: 32.3 ± 4.08	Mean 10.2 ± 6.8 years Insulin: 16.1% Blood glucose lowering tablets: 62.4% Insulin and tablets: 18.8% Lifestyle factors: 2.7%	Undergraduate: 20.1% Graduate: 57.5% Post-graduate: 22.4%
42, Lin et al., 2020 Taiwan Cross-sectional	n= 198 Means 51.2 ± 11 years 37% female	<24: 29.8% 24-26.9: 28.3% ≥27: 41.9% Total: 26.7 ± 4.6	<6 months: 15 (7.6%) 6 - 12 months: 28 (14.1%) 1 - 5 years: 86 (43.4%) 5 - 10 years: 38 (19.2%) >10 years: 31 (15.7%) Insulin: 5.1% Blood glucose lowering tablets: 27.8% Insulin and tablets: 57.5% Lifestyle factors: 9.1%	Secondary school (or lower): 49% University: 51%
43, Novak et al., 2017, United States Cross-sectional	n= 67 couples *Person with diabetes:* Mean 57 ± 9.8 years 43% female	Not reported	Mean 11 ± 9.2 years Management strategies not reported	*Person with diabetes:* Primary school: 0.9% Secondary school: 12.1% Technical training: 43.1% University: 21.6% Postgraduate: 22.4%
44, Shao et al., 2017, China Cross-sectional	n= 532 Mean 63 ± 16.7 years 57.1% female	Type D mean: 24.2 ± 3.7 Non-Type D mean: 24.1 ± 3.7	Not reported	Primary school: 47.8% Secondary school: 29.1% University: 23.1%
45, Skinner et al., 2014, Australia Cross-sectional	n= 1551 Mean 66 ± 11.1 years 47.1% female	Mean: 31.3 ± 6.2 <25: 13.3% 25-29: 32.3% ≥30: 54.4%	Median 9 years (IQR 2.9-15.6) Blood glucose-lowering tablets and/or insulin: 61.8%	Primary school or lower: 12%
46, Szymborska-Kajanek et al., 2006, Poland Cross-sectional	n= 41 Mean 57 ± 5.1 years 51% female	Mean 31.7 ± 6.0	8.6 ± 2.9 years Management strategies not reported	Not reported
31, Vergès et al., 2021 France Cross-sectional	N= 386 Mean 64.7 ± 10.4 years 39.4% female	Mean: 31.5 ± 6.5	14.2 ± 9.7 years Diabetes management strategies not reported	Not reported
32, Hall et al., 2009 Canada Prospective cohort	n= 204 Mean 58 ± 10.6 years, 59% female	<25: 10.1% 25-29.9: 33.3% 30-34.9, 24.2% ≥35: 31.3%	Mean 3 ± 1.8 months Blood glucose lowering tablets: 34.5%	Primary school: 10.4% Secondary school: 35.6% University: 54%
33, Davies et al., 2010 Australia Prospective Cohort	n= 74 Mean 61 ± 11.1 years 57% female	Not reported	Diabetes duration and management strategies not reported	Primary school: 37.9% Secondary school: 5.4% Technical training: 29.7% University: 27%
34, Helgeson et al., 2016 United States Prospective cohort	n= 70 couples *Person with diabetes* Mean 55 ± 9.8 years 51% female	Not reported	1.4 ± 1.1 years Insulin: 7% Blood glucose-lowering tablets: 63% Lifestyle factors: 11% Tablets and insulin: 19%	Median: University education
35, Li et al., 2016 China Prospective cohort	n= 412 Mean 57 ± 11.45 years 48% female	Total mean: 25.0 ± 3.2 Type D: 25.7 ± 3.1 Non-Type D: 24.7 ± 3.2	Mean 7.9 ± 5.5 years Management strategies not reported	Primary school: 11.2% Secondary school: 37% University: 51.8%
36, Nefs et al., 2012 Netherlands Prospective cohort	n= 1553 Mean 69 ± 10.2 years 52% female	Mean: 29.6 ± 4.6	Mean 6.3 ± 4.9 years Insulin: 0.9% Blood glucose-lowering tablets: 75.7% Tablets and insulin: 7.8% Lifestyle factors: 15.1% No treatment: 0.5%	Primary and/or secondary school: 63.5%
37, Sanatkar et al., 2020 Australia Prospective cohort	n= 199 Mean 60 ± 9.05 years ~55% female	Not reported	Not reported	Not reported
38, Fan et al., 2016 China Randomised Controlled Trial	n= 280 *Study group:* Mean 63 ± 10.72 years *Control group:* Mean 64.9 ± 10.14 years 43.6% female	*Study group* Baseline mean 24.3 ± 3.7 *Control group* Baseline mean 24.3 ± 3.9	*Study group* Mean 11.4 ± 4.8 years *Control group* Mean 11.6 ± 5 years Management strategies not reported	*Study group* Primary school: 58% Secondary school: 23.2% University: 18.8% *Control group* Primary school: 63.7% Secondary school: 20.3% University: 15.9%
39, Fisher et al., 2014 United States Randomised Controlled Trial	n= 392 *Overall mean:* 56 ± 9.55 years 54% female *Leap Ahead group mean*: 55 ± 10.9 years 59% female *Computer-assisted self-management group mean:* 57 ± 8.8 years 48% female *Computer-assisted self- management and problem-solving group mean:* 56 ± 9.4 years 56% female	*Overall mean:* 33.1 ± 7.8 *Leap Ahead group mean*: 33.3 ± 8.4 *Computer-assisted self-management group mean:* 32.1 ± 7.2 *Computer-assisted self- management and problem-solving group mean:* 33.9 ± 7.9	*Overall mean:* 6.9 ± 5.9 years *Leap Ahead group mean*: 7.6 ± 6.4 years *Computer-assisted self-management group mean:* 6.9 ± 6 years *Computer-assisted self- management and problem-solving group mean:* 6.5 ± 5.5 years Insulin *Overall:* 17.9% *Leap Ahead group:* 19.8% *Computer-assisted self-management group:* 15.3% *Computer-assisted self- management and problem-solving group:* 19.2%	*Overall* Primary and/or secondary school: 8.7% Technical training: 30.4% University: 61.0% *Leap Ahead group*: Primary and/or secondary school: 10.4% Technical training: 28.1% University: 61.5% *Computer-assisted self-management group:* Primary and/or secondary school: 8% Technical training: 30% University: 62% *Computer-assisted self- management and problem-solving group:* Primary and/or secondary school: 8.2% Technical training: 32.2% University: 59.6%

**Table 2:** Personality constructs, weight management indices and findings by indices.

Author, Year, Country, Study design	Personality construct (measurement tool)	Weight management indices (measurement tools)	Findings			
			Body Mass Index (BMI)	Adiposity	Physical activity	Healthy diet
40, Albargawi et al., 2016 Saudi Arabia Cross-sectional	Locus of control (Multidimensional Health Locus of Control Scale and the God Locus of Health Control Scale)	Physical activity and diet (Revised and Expanded Summary of Diabetic Self-Care Activity Scale, Arabic version)	Not measured	Not measured	Not significant	After controlling for sex and marital status: external health locus of control predicted less healthy diet: General diet: B= -0.371, p=0.05; Specific diet: B= -0.422, p=0.018
48, Elran-Barak et al., 2019 Israel Cross-sectional	Big Five (BFI-44)	Physical activity (3m-walk test, hand grip strength), BMI (assessment method not reported)	Not significant	Not measured	Neuroticism positively associated with 3m walk in seconds (r= 0.139, p<0.01) and negatively with hand grip strength (r= −0.191, p<0.001) Conscientiousness positively associated with hand grip strength (r= 0.12, p<0.05). Agreeableness positively associated with 3m walk in seconds (r= 0.11, p<0.05). Openness positively associated with hand grip strength (r= 0.11, p<0.05).	Not measured
41, Köbling et al., 2020 Hungary Cross-sectional	Type D (Hungarian version - modified DS14 utilising 10 items)	Physical activity, diet (each measured with a single item) and BMI (assessment tool not reported)	Not reported	Not measured	Type D personality associated with lower physical activity (r= -0.14, p<0.05)	Not reported
42, Lin et al., 2020 Taiwan Cross-sectional	Type D (DS14 – Taiwanese version)	Physical activity and diet (subscales of the Diabetes Self-Care Scale – Chinese version), BMI (assessment method not reported)	Not significant	Not measured	Type D personality associated with lower physical activity (t= 5.70, p<0.05)	Type D personality associated with less healthy diet (t= 15.50, p<0.001)
43, Novak et al., 2017 United States Cross-sectional	Neuroticism (subscale of BFI44), negative affect (subscale of the Positive & Negative Affect Schedule)	Physical activity and diet (subscales of the Summary of Diabetes Self-care Activities measure)	Not measured	Not measured	Direct: Neuroticism and negative affect not associated with physical activity (not significant) Indirect: Higher neuroticism was associated with lower physical activity (B=0.12, p = 0.001, 95%CI=-0.209, -0.064, R^2 =^ 0.27) *via* higher depressive symptoms and lower couple-level diabetes efficacy. A one standard deviation (SD) unit increase in neuroticism is associated with a 0.12 SD unit decrease in physical activity *via* the effect of neuroticism on depressive symptoms, and depressive symptoms on couple-level diabetes self-efficacy	Direct: Neuroticism associated with healthy diet r= -.23, p<.05. Negative affect associated with healthy diet r= -.38, p<.01 Indirect: Higher neuroticism was associated with lower healthy diet engagement B=0.17, p= 0.003, 95%CI = -0.264, -0.092 R^2 =^ 0.54) *via* higher depressive symptoms and lower couple-level diabetes efficacy. A one standard deviation (SD) unit increase in neuroticism is associated with a 0.17 SD unit decrease in healthy diet engagement *via* the effect of neuroticism on depressive symptoms, and depressive symptoms on couple-level diabetes self-efficacy
44, Shao et al., 2017 China Cross-sectional	Type D (DS14)	BMI, waist circumference, hip circumference, waist-to-hip ratio (self-report survey or medical records)	Not significant	Not significant (DS14 Total score) Negative affect subscale showed a significant negative relationship with waist-hip ratio (r=-.12, p<.01).	Not measured	Not measured
45, Skinner et al., 2014 Australia Cross-sectional	Big Five (BFI-44)	BMI (clinical examination)	An increase of 1 kg/m2 was associated with a 14% decrease in Conscientiousness (95% CI -20% to -8%) No other significant relationships between personality and BMI	Not measured	Not measured	Not measured
46, Szymborska-Kajanek et al., 2006 Poland Cross-sectional	Big Five (NEO-FFI)	BMI, waist-to-hip ratio (clinical examination)	Neuroticism was higher and conscientiousness lower for those with BMI over 30kg/m2 (obese range) No other significant relationships between personality and BMI	Not significant	Not measured	Not measured
31, Vergès et al., 2021 France Cross-sectional	Type A (Bortner Scale)	BMI (assessment tool not reported)	Not significant	Not measured	Not measured	Not measured
32, Hall et al., 2009 Canada Prospective cohort	Anxious temperament (Behavioural Inhibition/Behavioural Approach Scales)	Physical activity (Physical Activity Scale for the Elderly [PASE]), diet (National Cancer Institute Fat Screener) and BMI	Not significant	Not measured	Negative relationship between anxiety and physical activity B=-0.179, p<0.01 at follow-up	Not significant
33, Davies et al., 2010 Australia Prospective cohort	Big Five (IPIP-NEO-50)	Physical activity (Godin leisure-time exercise questionnaire modified to assess 14-day period)	Not measured	Not measured	Conscientiousness positively correlated with physical activity: r=0.37 p<0.01.	Not measured
34, Helgeson et al., 2016 United States Prospective cohort	Unmitigated communion (Unmitigated Communion Scale)	Physical activity (one item - Did you exercise today? [no/yes]), diet (one item - How much did you follow your diet today? [1=not at all, 5 = very much])	Not measured	Not measured	Not significant	Participants higher in unmitigated communion reported more sub-optimal diet consistency (R^2^=-0.22, p=0.04).
35, Li et al., 2016 China Prospective cohort	Type D (DS14 – Chinese version)	Physical activity (assessment tool not reported), BMI (medical records)	Participants with higher scores of type D personality had a significantly higher BMI (t=2.37, p=0.009)	Not measured	Not reported	Not measured
36, Nefs et al., 2012 Netherlands Prospective cohort	Type D (DS14)	Physical activity (assessment tool not reported), BMI (clinical examination)	Not significant	Not measured	In women only, Type D personality was associated with less “active” health behaviour, which relates to daily activities of mild-to-moderate intensity for at least 2 hours per week	Not measured
37, Sanatkar et al., 2020 Australia Prospective cohort	Big Five (BFI-44)	Physical activity and diet (subscales of the Self-Management Profile for Type 2 Diabetes (SMP-T2D)	Not measured	Not measured	Conscientiousness was positively correlated with physical activity: r=0.15 p<0.05.	Conscientiousness was positively correlated with healthy diet: r=0.31 p<0.01 Neuroticism was negatively correlated with healthy diet: r= -0.20, p<0.01.
38, Fan et al., 2016 China Randomised Controlled Trial	Extraversion and neuroticism (Eysenck Personality Questionnaire)	BMI, waist circumference (clinical examination)	Individualised diabetes education tailored to the participants’ personality was associated with a greater reduction in BMI (d=1.05, p=0.002) than usual care (non-individualised education)	Individualised diabetes education tailored to the participants’ personality was associated with a greater reduction in waist circumference (d=0.28, p=0.032) than usual care (non-individualised education)	Not measured	Not measured
39, Fisher et al., 2014 United States Randomised Controlled Trial	Conscientiousness (9-item sub-scale)	Physical activity Community Health Activity Program for Seniors [CHAMPS]), Diet (National Cancer Institute Percent Energy from Fat Screener), BMI (assessment method not reported)	Not reported (collected)	Not measured	Those with both high conscientiousness and self-efficacy at baseline showed the largest increases in physical activity (F=4.43 p= 0.04)	Not significant

A narrative synthesis of the findings was conducted, focusing on the relationship between personality and weight management.

### 2.5 Assessment of risk of bias

Quality of studies was evaluated using the Joanna Briggs Institute’s (JBI) critical appraisal checklists (47) appropriate to each study type. Studies were not excluded based on the quality rating received. Each study was assessed by the first author, with 50% of studies also assessed by the fourth author. Conflicts were resolved through discussion and, where required, in consultation with a third author. Studies were rated across between five and eight domains, depending on the study design, as being a). low risk of bias, b). some concerns, c). high risk of bias, or, d). no information/not applicable. The JBI critical appraisal checklist guidance does not specify any aggregated calculation methodology for a study’s overall rating. As such, risk of bias is assessed and discussed in terms of the number of studies, and the individual domains of bias, that were assessed at a certain rating (refer to section 3.3).

## 3 Results

**Figure 1:**
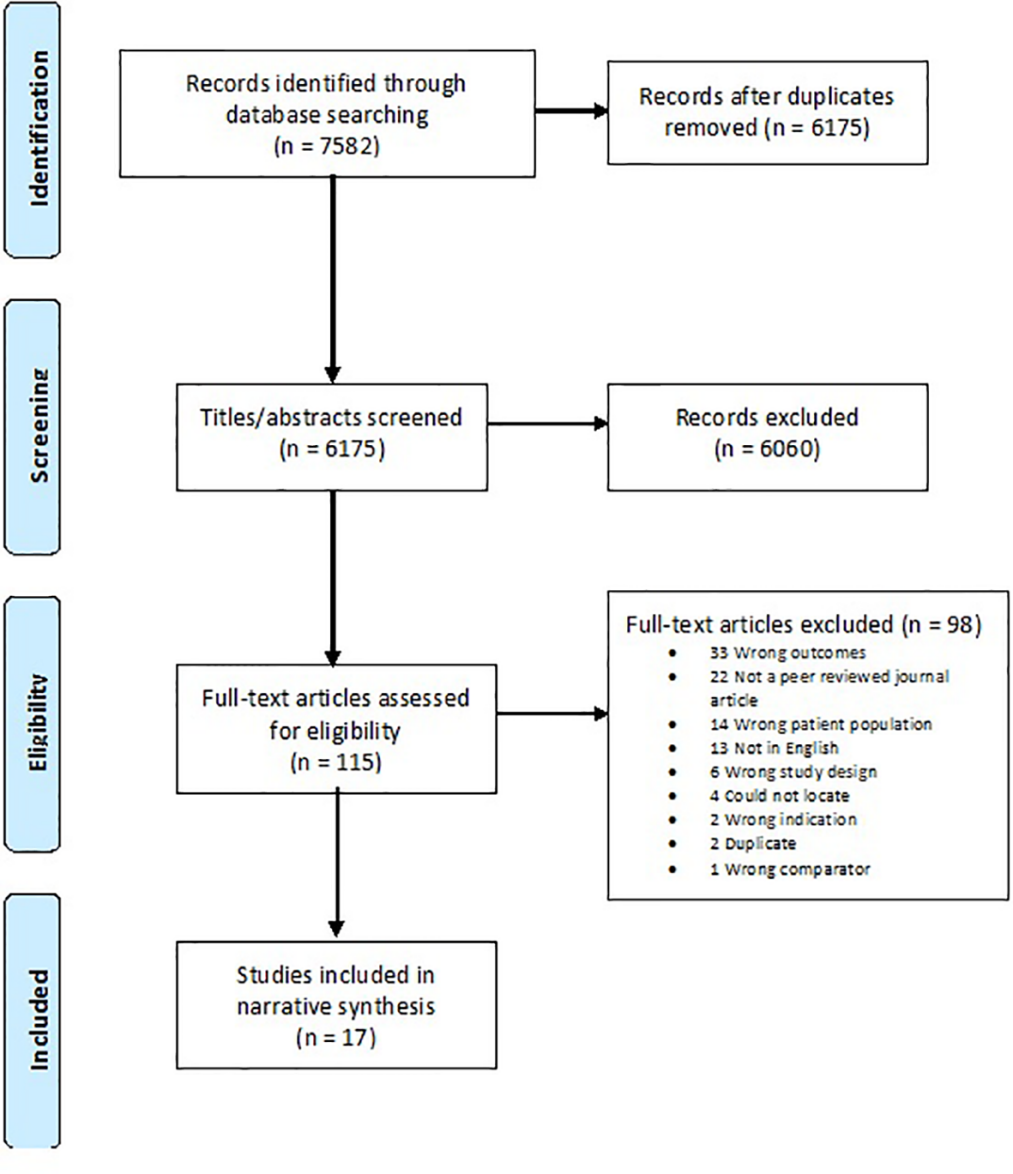
PRISMA Flowchart.

**Figure 1** displays the PRISMA flowchart of the systematic search. After removing duplicates, 6,175 titles and abstracts were screened. Of the 115 full texts assessed for inclusion, 98 were excluded and k=17 studies met the inclusion criteria.

### 3.1 Study and sample characteristics

Study and sample characteristics are reported in **Table 1**. Most (15/17; 88%) of the included studies were published since 2010. Included studies comprised nine cross-sectional studies (31, 40–46, 48), six prospective cohort studies (32–37) and two randomised controlled trials (RCTs) (38, 39). Study duration for cohort and RCTs ranged between two weeks and 12 months. For cohort studies, follow-up data were used for analysis and synthesis except for Li et al. (2016) (35), where baseline data were used due to the different primary outcome measured. For both RCTs (38, 39), between-groups differences at follow-up were analysed. Studies were conducted across eleven countries, with three studies each in Australia (33, 37, 45), China (35, 38, 44), and the USA (34, 39, 43), and one study each per other country (31, 32, 36, 40, 41, 42, 46, 48). The total combined sample size of included studies was N=6,672, and the sample size of individual studies ranged from N=30 to N=1,553.

Where reported (k=9), average diabetes duration (7.8 years) varied widely across studies, from recently diagnosed (3 ± 2 months [32)] to long-standing diabetes [14 ± 10 years (31)]. Current diabetes treatment was reported in seven studies (32, 34, 36, 39, 41, 42, 45), and included oral glucose-lowering medications (range: 28-76%) and insulin injections (range: 0.9% to 18%). Eleven studies reported participants’ BMI (31, 35, 36, 38, 39, 41, 42, 44–46, 48), which ranged from 24.1 ± 3.7kg/m^2^ to 33.1 ± 7.8kg/m^2^. In the study where BMI categories were reported only (32), 55.5% of participants had a BMI ≥30kg/m^2^. One study (41) specified BMI as part of its inclusion criteria (BMI of 25–45 kg/m^2^), one specified newly diagnosed participants (32), and one specified concurrent mild-to-moderate depression (37).

### 3.2 Measurement of personality and weight management outcomes

**Table 2** displays the personality and weight management constructs, questionnaires and/or indices reported by each study. Regarding the investigation of personality, eight studies measured one or more of the Big Five domains [k=5 assessed five domains (33, 37, 45, 46, 48), k=1 assessed two domains *via* the Eysenck Personality Inventory (38), and k=2 assessed a single domain (39, 43)]. Five studies (35, 36, 41, 42, 44) assessed Type D personality, measured by the Type D Scale-14 (DS14), and one (43) incorporated the negative affect subscale of the Type D personality measure. One study each assessed: Type A personality (31) (*via* the Bortner Rating Scale), anxious temperament (32) (*via* the Behavioral Inhibition and Behavioral Approach Scales), locus of control (40) (*via* the Multidimensional Health Locus of Control questionnaire), and unmitigated communion (34) (*via* the Unmitigated Communion scale).

Regarding weight management measures, five studies examined behaviours alone, five assessed outcomes alone, and seven studies examined both. Behaviours examined included physical activity (k=12) and healthy diet (k=8). Physical activity was assessed *via* validated self-report measures [k=7 (32, 33, 37, 39, 40, 42, 43)], unvalidated self-report measures [k=2 (34, 41)], and physical capacity testing [k=1 (48)], while two studies (35, 36) did not report the assessment tool used. Healthy diet was assessed *via* validated [k=6 (32, 37, 39, 40, 42, 43)] or unvalidated [k=2 (34, 41)] self-report measures. Weight management outcomes included a) BMI (k=12) collected *via* medical records [k=2 (35, 44)], clinical exam [k=4 (36, 38, 45, 46)], self-report [k=1 (32)], or unknown assessment method [k=5 (31, 39, 41, 42, 48)]; and b) adiposity (k=3), including waist-to-hip ratio [k=2 (44, 46)], waist circumference [k=2 (38, 44)], or hip circumference), [k=1 (44)].

### 3.3 Risk of Bias

**Figure 2:**
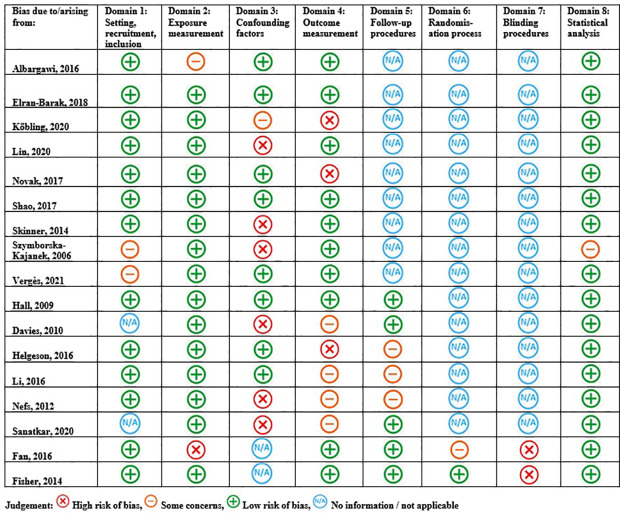
Risk of bias and quality assessments for included studies.

**Figure 2** displays the study quality and risk of bias assessment for the identified studies. Overall, k=3 (32, 44, 48)/17 studies were rated as having a low risk of bias across all risk domains relevant to their study design and a further k=5 (39, 40, 42, 43, 45) studies were rated as having only one relevant risk domain of some concern or high risk. The area of least concern was statistical analysis (Domain 8: k=16 (31–45, 48)/17 low risk of bias). Potential bias mostly related to the lack of identification, and mitigation of, confounding variables (Domain 3: k=6 (33, 36, 37, 42, 45, 46)/17 some concern or high risk of bias). Outcome measurement also posed potential bias (Domain 4: k=8 (31, 34–36, 39, 41, 42, 48)/17 some concern or high risk of bias), whereby single-item and/or unvalidated physical activity and healthy diet questions were employed (k=2 (34, 41)/12) or assessment tools used for weight management outcomes were not reported (k=5 (31, 39, 41, 42, 48): BMI; k=2 (35, 36): physical activity).

### 3.4 Evidence synthesis

**Table 3** summarises the associations between personality traits and weight management indicators. In summary, across eight studies assessing one or more Big Five domains (33, 37–39, 43, 45, 46, 48), weak-to-moderate significant associations were observed between an indicator of weight management and neuroticism (k=5 (37, 38, 43, 46, 48)/7, health compromising associations), conscientiousness (k=6 (33, 37, 39, 45, 46, 48)/6, health enhancing associations), and extraversion (k=1 (38)/6, health enhancing association), openness (k=1 (48)/5, health enhancing association) and agreeableness (k=1 (48)/5, health compromising association). In addition, all studies assessing Type D personality (k=5 (35, 36, 41, 42, 44)/5), negative affect (k=1 (43)/1), anxious temperament (k=1 (32)/1), locus of control (k=1 (40)/1) and unmitigated communication (k=1 (34)/1) identified weak-to-moderate significant health compromising associations with at least one weight management indicator of interest. Only the study investigating Type A personality (31) observed no relationship with weight management.

**Table 3:** Summary of the assessment of personality and the significance and direction of associations with weight management indices.

Author, Year	Personality construct										Weight management indices^¤^				
	Anxious Temp.	Locus of Control	Unmitigated Communion	Type A	Type D	N	E	O	C	A		BMI	Adiposity	Physical Activity	Healthy Diet
32, Hall et al., 2009	X											NS		–	NS
40, Albargawi et al., 2016		X												NS	–
34, Helgeson et al., 2016			X											NS	–
31, Vergès et al., 2021				X								NS			
41, Köbling et al., 2020					X							NR		–	NR
35, Li et al., 2016					X							+		NR	
42, Lin et al., 2020					X							NS		–	–
36, Nefs et al., 2012					X							NS		–	
44, Shao et al., 2017					X							NS	–		
33, Davies et al., 2010						X	X	X	X*	X				+	
48, Elran-Barak et al., 2019						X*	X	X*	X*	X*		NS		^+N (3m walk)^ ^-N (hand grip)^ ^+C hand grip)^ ^+A (3m walk)^ ^+O hand grip)^	
45, Skinner et al., 2014						X	X	X	X*	X		–			
37, Sanatkar et al., 2020						X*	X	X	X*	X				+^C^	+^C^ -^N^
46, Szymborska-Kajanek et al., 2006						X*	X	X	X*	X		+^N^ - ^C^	NS		
43, Novak et al., 2017					X*_NA_	X*								NS	-^N^ -^NA^
38, Fan et al., 2016						X*	X*					GD	GD		
39, Fisher et al., 2014									X*			NR		+	NS

Outcome measures do not imply a primary outcome of the study, X* signifies which personality factor demonstrated the significant association (if more than one tested), N Neuroticism, E Exraversion, O Openness, C Conscientiousness, Agreeableness, X_NA_ Negative Affect subscale, - signifies a negative association, + signifies a positive association, NS: finding not significant, NR: finding not reported, -^NA^: negative association with negative affect, +N positive association with Neuroticism, -N negative association with Neuroticism, +C positive association with Conscientiousness, -C negative association with Conscientiousness, +A positive association with Agreeableness, +O positive association with Openness, GD group differences found

#### 3.4.1 Personality and physical activity

Eight of twelve studies investigating the relationship between personality and physical activity reported significant associations (32, 33, 36, 37, 39, 41, 42, 48). Specifically, regarding Big Five personality traits, k=4 (33, 37, 39, 48)/4 studies found weak-to-moderate health enhancing associations between physical activity and conscientiousness. Additionally, k=1 (48)/4 studies identified weak health compromising associations with neuroticism and agreeableness and a health enhancing association with openness and physical activity. Three (36, 41, 42) of the five studies that investigated the relationship between Type D personality or negative affect and physical activity reported a weak significant negative association. However, Nefs et al. (36) reported that this association was observed only for women. Also, whilst not finding significant direct effects of neuroticism on physical activity, Novak et al. (43) did find that higher levels of neuroticism were associated with lower levels of physical activity through depressive symptoms and couple-level diabetes efficacy. A weak, but significant, negative association with physical activity was also reported by the single study examining anxious temperament (32). The studies assessing locus of control (40) and unmitigated communion (34) found no associations with physical activity.

#### 3.4.2 Personality and healthy diet

Eight studies (32, 34, 37, 39–43) investigated the relationship between personality and healthy diet, of which five identified significant associations (34, 37, 40, 42, 43). Specifically, k=2 (43) (37)/3 studies utilising the Big Five personality traits found that healthy diet was positively and moderately associated with conscientiousness and negatively, weakly, associated with neuroticism. Weak-to-moderate negative relationships with healthy diet were also reported for Type D personality (k=1 (42)/2), negative affect (k=1 (43)/1), locus of control (k=1 (43)/1), and unmitigated communion (k=1 (34)/1). The single study examining anxious temperament (32) did not find a significant relationship with healthy diet.

#### 3.4.3 Personality, body mass index and adiposity

Overall, five studies used the Big Five to examine the relationship between personality and BMI (38, 39, 45, 46, 48) and adiposity (38, 46) with three studies identifying significant associations. A weak-to-moderate negative association was observed between conscientiousness and BMI in k=2 (45, 46)/4 studies, and a moderate, positive relationship was observed between neuroticism and BMI in k=1 (46)/4 studies. The RCT study (38) assessing extraversion and neuroticism found that tailoring the treatment group’s intervention based on personality structure had a strong beneficial between groups effect on BMI and a weak beneficial between groups effect on waist circumference.

Of the k=5 (35, 36, 41, 42, 44) studies assessing Type D personality and BMI, k=1 (35) found a weak positive relationship. The k=1 (44) study to examine the association between Type D personality and adiposity found a weak, but significant, negative relationship between waist-to-hip ratio and the negative affect subscale. There was no significant association between Type A personality (k=1) (31), nor anxious temperament (k=1) (32) and BMI.

## 4 Discussion

This is the first systematic review to examine the association between personality and weight management in adults with type 2 diabetes. Neuroticism, conscientiousness, extraversion, openness, agreeableness, Type D personality (negative affect and social inhibition), anxious temperament, locus of control and unmitigated communion, all displayed relationships across indices of physical activity, healthy diet, BMI and adiposity. Specifically, personality traits characterising negative emotionality were associated with sub-optimal weight management, while conscientiousness was associated with more optimal weight management. None of the 17 studies had the primary aim of investigating the association between personality and weight management. There was substantial heterogeneity, in terms of aims, study designs, and measurement.

Across identified studies, personality was most commonly operationalised using the Big Five (15), which has emerged as the most consistent representation of personality over the last 30 years. Relating other personality constructs to the Big Five can therefore provide a robust and well accepted conceptualisation of the relationship between personality and weight management in adults with type 2 diabetes. Anxious temperament, or anxiety, is a facet of the neuroticism domain (49) and Type D personality has been shown to represent neuroticism and reversed extraversion (50). Elements of neuroticism, extraversion (reversed) and (low) conscientiousness have been found to represent locus of control (51–54), whereas unmitigated communion shares features of agreeableness and neuroticism (55, 56).

Considering review findings through a Big Five lens, the personality constructs influencing weight management in adults with type 2 diabetes relate to neuroticism (including from Type D personality, external locus of control and anxious temperament), conscientiousness, and (to some degree) extraversion, openness and agreeableness (including from unmitigated communion). However, examining only those studies that specifically assessed the Big Five traits suggests there is limited support for extraversion, openness and agreeableness. In the case of extraversion, whilst more frequently associated with weight management in the general population personality literature, it is often conflicting in terms of its health enhancing or compromising influence (12). Regarding openness and agreeableness, the general population personality literature has less frequently associated these traits with weight management (12). Our review suggests that neuroticism and conscientiousness have consistent associations with weight management among adults with type 2 diabetes, with limited and less consistent associations with extraversion, openness and agreeableness, as observed in studies of cardiovascular disease (24).

Despite the literature’s conflicting evidence of extraversion’s health enhancing and compromising relationships, the greater social confidence linked to extraversion has been associated with healthcare attendance (57). Our findings indicate that the social self-efficacy and support structures that extraversion foster may be associated with health protective weight management behaviours and outcomes. The impact of spousal support dynamics in two of the studies (34, 43) also demonstrated the involvement of neuroticism and agreeableness in weight management behaviours. The latter study providing evidence of a mechanism for how reduced self-care, including the negative impact this has on weight management behaviours, is manifested through personality and traits that prioritise others, as is the case in unmitigated communion.

While few studies have examined the role of personality in weight management for type 2 diabetes, there is more extensive evidence for the role of self-efficacy and diabetes distress (10, 58). Two studies included in this review demonstrated the conceptual similarities, and interaction between, self-efficacy and conscientiousness (39), as well as diabetes distress and neuroticism (37), and their respective optimal and sub-optimal relationships with weight management. Therefore, personality may explain a person’s capacity for maintaining optimal levels of diabetes self-efficacy and/or their experience of diabetes distress, and the subsequent influence on weight management behaviours. Indeed, previous research has identified neuroticism’s involvement in the experience of negative emotions and their association with sub-optimal glucose outcomes (59) as well as medication taking behaviour (60). With regard to self-efficacy, the organisation, planning and discipline that define the trait conscientiousness have been shown to be associated with optimal performance and coping with daily regimens and routines such as foot checking behaviour (61), glucose monitoring (37) as well as HbA1c outcomes (62).

In contrast with cardiovascular disease, diabetes research regarding personality’s relationship with weight management is in its infancy. Despite this long history of cardiovascular disease-based personality research, with replicated findings of association (24), there are few applied examples of personality-informed interventions in cardiovascular disease research. Perhaps progressively in this regard, one diabetes study (38) included in this review conducted a diabetes education intervention with content tailored to the personality of participants with positive results, suggesting further research is warranted. There are calls for a more individualised approach to clinical care and diabetes management based on personal traits, skills and education (38, 39). Yet, personality-weight management research in type 2 diabetes is limited. With the specific pharmacological and hormonal weight management challenges unique to diabetes, it is therefore important to establish any consistent personality-weight management relationships so that personality-informed management can advance.

The utility of evaluating personality within a clinical setting for routine behaviour change counselling is unclear, particularly given consultations are unlikely to allow for comprehensive measurement due to time constraints. Inclusion within diabetes education programs may be more feasible. For example, Fan et al. (38) tailored a diabetes education program to the personality of participants. By personalising the detail and emphasis regarding self-care plans, targets, involvement of family members, information on complications, medications and device use depending on participants’ trait configuration, weight management indicators improved. Understanding for whom novel interventions will most likely be effective for is an important component that personality assessment may be able to address. A long-standing view of personality was that it was unmalleable (63). But more recent research has found personality can change over time (63) and that bidirectional relationships exist between personality and weight management (64), which opens up new directions for personality-informed interventions and research.

Several limitations of the included studies should be noted. Whilst an inclusion criterion was the use of a validated personality inventory, comprehensive personality assessment was limited in some studies, e.g. short-forms, single domains, or measures that do not provide a full assessment of personality. The use of unvalidated, single-item weight management measures also reduced the validity of the findings, as did the reliance of several studies on self-reported BMI, diet and physical activity levels for which objective measures are available. Further, several studies did not address potentially confounding factors, which may alter the true strength of association between personality with weight management. For example, heterogeneity in study duration, diabetes duration and diabetes management strategies, all of which can influence weight management outcomes in isolation, may further complicate the role of confounding factors further. Comparisons across studies were also complicated by varied operationalisation of personality constructs, and together with the disparity in independent variables included, meant meta-analysis was not possible. The weak-to-moderate effect sizes reported across studies in this review should therefore be interpreted with these limitations in mind until research in this area expands and direct comparisons can be made. Finally, a certain degree of publication bias may also apply to this review with respect to the inclusion of certain studies and the interpretation of the outlined criteria.

A key strength of this review is the methodological rigour used including the five databases searched and the involvement of the lead author across the entire screening, data extraction and quality appraisal process, assisted by other members of the team. Broad inclusion criteria without limiters on personality assessment, weight management, diabetes complications or other comorbidities was also a strength, ensuring studies with differing primary objectives were identified for inclusion. Whilst an extensive systematic search was completed, the review may be limited by the exclusion of relevant studies that were not published in English.

Weight management is an important component of type 2 diabetes self-management. This novel systematic review identified 17 studies among adults with type 2 diabetes, with evidence emerging for weak-to-moderate relationships between weight management and the personality constructs of neuroticism and conscientiousness. Such findings are consistent with the conclusions drawn in general and cardiovascular disease populations regarding the role of personality in health behaviours and/or weight outcomes. However, despite the unique weight management challenges in diabetes, it is evident that the personality-weight management relationship in type 2 diabetes is under-researched. Further investigation is warranted in which the primary aim focuses on this relationship, and a comprehensive assessment of personality and weight management is undertaken, employing validated self-report measures and objective assessment of health behaviours and weight outcomes.

## Data availability statement

The original contributions presented in the study are included in the article/**Supplementary Material**. Further inquiries can be directed to the corresponding author.

## Author contributions

RG conceived of the research question, completed all abstract and full text screening, data extraction, quality assessment and initial manuscript development. EJK, CE, EH-T and JS contributed to screening of abstracts, CE and EJK contributed to full text screening, data extraction and quality assessment. All authors contributed to the article and approved the submitted version.

## Funding

RG is supported by a Deakin University Industry PhD Scholarship, in collaboration with AstraZeneca Australia (unrestricted educational grant). JS and EH-T are supported by core funding of The Australian Centre for Behavioural Research in Diabetes (ACBRD) provided by the collaboration between Diabetes Victoria and Deakin University. This study received funding from AstraZeneca Australia. The funder was not involved in the study design, collection,analysis, interpretation of data, the writing of this article or the decision to submit it for publication.

## Acknowledgments

The preliminary results of this study were presented at the Australasian Diabetes Congress (August 2021) and published in abstract form. No other results re-ported in this manuscript are published elsewhere.

## Conflict of interest

The authors declare that the research was conducted in the absence of any commercial or financial relationships that could be construed as a potential conflict of interest.

## Publisher’s note

All claims expressed in this article are solely those of the authors and do not necessarily represent those of their affiliated organizations, or those of the publisher, the editors and the reviewers. Any product that may be evaluated in this article, or claim that may be made by its manufacturer, is not guaranteed or endorsed by the publisher.

## References

[1] Gatineau M HC Holman N Outhwaite H Oldridge L Christie A Ells L . Adult obesity and type 2 diabetes. Oxford, England: Public Health England (2014).

[2] Ventura A Browne J Holmes-Truscott E Hendrieckx C Pouwer F Speight J . Diabetes MILES-2 2016 survey report. Melbourne: Diabetes Victoria (2016).

[3] Mogre V Johnson NA Tzelepis F Shaw JE Paul C . A systematic review of adherence to diabetes self-care behaviours: Evidence from low- and middle-income countries. J. Adv Nurs. (2019) 75(12):3374–89. doi: 10.1111/jan.14190 31453637

[4] The Royal Australian College of General Practitioners . Management of type 2 diabetes: A handbook for general practice. (East Melbourne, Victoria: RACGP). (2020)

[5] American Diabetes Association . 8. obesity management for the treatment of type 2 diabetes: Standards of medical care in diabetes. Diabetes Care (2021) 44(Supplement 1):S100–S10. doi: 10.2337/dc21-S008 33298419

[6] Blüher M . Obesity: global epidemiology and pathogenesis. Nat Rev Endocrinol. (2019) 15(5):288–98. doi: 10.1038/s41574-019-0176-8 30814686

[7] Van Gaal L Scheen A . Weight management in type 2 diabetes: Current and emerging approaches to treatment. Diabetes Care (2015) 38(6):1161–72. doi: 10.2337/dc14-1630 25998297

[8] Markowitz S Friedman MA Arent SM . Understanding the relation between obesity and depression: causal mechanisms and implications for treatment. Clin Psychol: Sci Pract (2008) 15(1):1–20. doi: 10.1111/j.1468-2850.2008.00106.x

[9] Pontiroli AE Miele L Morabito A . Increase of body weight during the first year of intensive insulin treatment in type 2 diabetes: systematic review and meta-analysis. Diabetes Obes Metab (2011) 13(11):1008–19. doi: 10.1111/j.1463-1326.2011.01433.x 21645195

[10] Nanayakkara N Pease A Ranasinha S Wischer N Andrikopoulos S Speight J . Depression and diabetes distress in adults with type 2 diabetes: results from the Australian national diabetes audit (ANDA) 2016. Sci Rep (2018) 8:1–10. doi: 10.1038/s41598-018-26138-5 PMC5959930 29777153

[11] Dutton GR Tan F Provost BC Sorenson JL Allen B Smith D . Relationship between self-efficacy and physical activity among patients with type 2 diabetes. J Behav Med (2009) 32(3):270–7. doi: 10.1007/s10865-009-9200-0 19156510

[12] Gerlach G Herpertz S Loeber S . Personality traits and obesity: A systematic review. Obes Rev (2015) 16(1):32–63. doi: 10.1111/obr.12235 25470329

[13] Corr PJ Matthews G . The Cambridge handbook of personality psychology. Cambridge, UK: Cambridge University Press (2009).

[14] Anglim J Horwood S Smillie LD Marrero RJ Wood JK . Predicting psychological and subjective well-being from personality: A meta-analysis. Psychol Bull (2020) 146(4):279. doi: 10.1037/bul0000226 31944795

[15] Goldberg LR . The development of markers for the big-five factor structure. Psychol Assess (1992) 4(1):26. doi: 10.1037/1040-3590.4.1.26

[16] Rotter JB . Generalized expectancies for internal versus external control of reinforcement. Psychol monographs: Gen Appl (1966) 80(1):1. doi: 10.1037/h0092976 5340840

[17] Norman P Bennett P Smith C Murphy S . Health locus of control and health behaviour. J Health Psychol (1998) 3(2):171–80. doi: 10.1177/135910539800300202 22021357

[18] Diehl M Owen SK Youngblade LM . Agency and communion attributes in adults’ spontaneous self-representations. Int J Behav Dev (2004) 28(1):1–15. doi: 10.1080/01650250344000226 18592013 PMC2441921

[19] Bakan D . The duality of human existence: An essay on psychology and religion. (Skokie, Illinois: Rand McNally & Co) (1966).

[20] Fritz HL Helgeson VS . Distinctions of unmitigated communion from communion: self-neglect and overinvolvement with others. J Pers Soc Psychol (1998) 75(1):121. doi: 10.1037/0022-3514.75.1.121 9686454

[21] Friedman M Rosenman RH . Type a behavior and your heart. 1st ed. (New York City: Knopf Doubleday Publishing Group) (1974) 276.

[22] Yoder L . Modifying the type a behavior pattern. J Relig Health (1987) 26(1):57–72. doi: 10.1007/BF01533295 24301840

[23] Denollet J Rombouts H Gillebert T Brutsaert D Sys S Stroobant N . Personality as independent predictor of long-term mortality in patients with coronary heart disease. Lancet (1996) 347(8999):417–21. doi: 10.1016/S0140-6736(96)90007-0 8618481

[24] Ati NAL Paraswati MD Wihastuti TA Utami YW Kumboyono K . The roles of personality types and coping mechanisms in coronary heart disease. A Syst. Rev (2020) 14(1):499–506.

[25] Conti C Carrozzino D Patierno C Vitacolonna E Fulcheri M . The clinical link between type d personality and diabetes. Front Psychiatry (2016) 7:113. doi: 10.3389/fpsyt.2016.00113 27445869 PMC4914509

[26] Jokela M Elovainio M Nyberg ST Tabák AG Hintsa T Batty GD . Personality and risk of diabetes in adults: Pooled analysis of 5 cohort studies. Health Psychol (2014) 33(12):1618–21. doi: 10.1037/hea0000003 23957901

[27] Phillips AC Batty GD Weiss A Deary I Gale CR Thomas GN . Neuroticism, cognitive ability, and the metabolic syndrome: The Vietnam experience study. J Psychosom Res (2010) 69(2):193–201. doi: 10.1016/j.jpsychores.2010.01.016 20624519

[28] Oshio A Taku K Hirano M Saeed G . Resilience and big five personality traits: A meta-analysis. Pers Individ Dif (2018) 127:54–60. doi: 10.1016/j.paid.2018.01.048

[29] Alonso-Tapia J Rodríguez-Rey R Garrido-Hernansaiz H Ruiz M Nieto C . Coping, personality and resilience: Prediction of subjective resilience from coping strategies and protective personality factors. Behav Psychol/Psicología (2019) 27(3):375–389.

[30] Page MJ McKenzie JE Bossuyt PM Boutron I Hoffmann TC Mulrow CD . The PRISMA 2020 statement: An updated guideline for reporting systematic reviews. Syst Rev (2021) 10(1):1–11. doi: 10.1136/bmj.n71 33781348 PMC8008539

[31] Vergès B Brands R Fourmont C Petit J-M Simoneau I Rouland A . Fewer type a personality traits in type 2 diabetes patients with diabetic foot ulcer. Diabetes Metab (2021) 47(6):101245. doi: 10.1016/j.diabet.2021.101245 33722768

[32] Hall PA Rodin GM Vallis TM Perkins BA . The consequences of anxious temperament for disease detection, self-management behavior, and quality of life in type 2 diabetes mellitus. J Psychosom Res (2009) 67(4):297–305. doi: 10.1016/j.jpsychores.2009.05.015 19773022

[33] Davies CA Mummery WK Steele RM . The relationship between personality, theory of planned behaviour and physical activity in individuals with type II diabetes. Br J sports Med (2010) 44(13):979–84. doi: 10.1136/bjsm.2008.050930 18753158

[34] Helgeson VS Mascatelli K Seltman H Korytkowski M Hausmann LRM . Implications of supportive and unsupportive behavior for couples with newly diagnosed diabetes. Health Psychol (2016) 35(10):1047–58. doi: 10.1037/hea0000388 PMC5033701 27280364

[35] Li X Zhang S Xu H Tang X Zhou H Yuan J . Type d personality predicts poor medication adherence in Chinese patients with type 2 diabetes mellitus: A six-month follow-up study. PloS One (2016) 11(2):e0146892. doi: 10.1371/journal.pone.0146892 26894925 PMC4760773

[36] Nefs G Pouwer F Pop V Denollet J . Type D (distressed) personality in primary care patients with type 2 diabetes: validation and clinical correlates of the DS14 assessment. J Psychosom Res (2012) 72(4):251–7. doi: 10.1016/j.jpsychores.2012.01.006 22405217

[37] Sanatkar S Baldwin P Clarke J Fletcher S Gunn J Wilhelm K . The influence of personality on trajectories of distress, health and functioning in mild-to-moderately depressed adults with type 2 diabetes. Psychol Health Med (2020) 25(3):296–308. doi: 10.1080/13548506.2019.1668567 31537118

[38] Fan MH Huang BT Tang YC Han XH Dong WW Wang LX . Effect of individualized diabetes education for type 2 diabetes mellitus: a single-center randomized clinical trial. Afr. Health Sci (2016) 16(4):1157–62. doi: 10.4314/ahs.v16i4.34 PMC5398463 28479909

[39] Fisher L Hessler D Masharani U Strycker L . Impact of baseline patient characteristics on interventions to reduce diabetes distress: the role of personal conscientiousness and diabetes self-efficacy. Diabetic Med (2014) 31(6):739–46. doi: 10.1111/dme.12403 PMC4028368 24494593

[40] Albargawi M Snethen J Gannass AA Kelber S . Perception of persons with type 2 diabetes mellitus in Saudi Arabia. Int J Nurs Sci (2016) 3(1):39–44. doi: 10.1016/j.ijnss.2016.02.007

[41] Köbling T Váradi Z Katona E Somodi S Kempler P Páll D . Predictors of dietary self-efficacy in high glycosylated hemoglobin A1c type 2 diabetic patients. J Int Med Res (2020) 48(6):0300060520931284. doi: 10.1177/0300060520931284 32588697 PMC7325457

[42] Lin Y-H Chen D-A Lin C Huang H . Type d personality is associated with glycemic control and socio-psychological factors on patients with type 2 diabetes mellitus: A cross-sectional study. Psychol Res Behav Manage (2020) 13:373. doi: 10.2147/PRBM.S245226 PMC7200262 32431557

[43] Novak JR Anderson JR Johnson MD Hardy NR Walker A Wilcox A . Does personality matter in diabetes adherence? exploring the pathways between neuroticism and patient adherence in couples with type 2 diabetes. Appl Psychol Health well-being (2017) 9(2):207–27. doi: 10.1111/aphw.12087 PMC5511078 28401663

[44] Shao Y Yin H Wan C . Type d personality as a predictor of self-efficacy and social support in patients with type 2 diabetes mellitus. Neuropsychiatr Dis Treat (2017) 13:855–61. doi: 10.2147/NDT.S128432 PMC5365332 28360523

[45] Skinner TC Bruce DG Davis TM Davis WA . Personality traits, self-care behaviours and glycaemic control in type 2 diabetes: The fremantle diabetes study phase II. Diabetic Med (2014) 31(4):487–92. doi: 10.1111/dme.12339 24147848

[46] Szymborska-Kajanek A Wróbel M Cichocka M Grzeszczak W Strojek K . The assessment of influence of personality type on metabolic control and compliance with physician's instruction in type 2 diabetic patients. Exp Clin Diabetol. / Diabetol Doswiadczalna i Kliniczna (2006) 6(1):11–5.

[47] Moola S MZ Tufanaru C Aromataris E Sears K Sfetcu R Currie M . Chapter 7: Systematic reviews of etiology and risk. In: Aromataris E Munn Z , editors. JBI manual for evidence synthesis. (Adelaide, South Australia: JBI) (2020). Available at: https://synthesismanual.jbi.global.

[48] Elran-Barak R Weinstein G Beeri MS Ravona-Springer R . The associations between objective and subjective health among older adults with type 2 diabetes: The moderating role of personality. J psychosom Res (2019) 117:41–7. doi: 10.1016/j.jpsychores.2018.12.011 30665595

[49] Costa PT Jr. McCrae RR Kay GG . Persons, places, and personality: Career assessment using the revised NEO personality inventory. J Career Assess (1995) 3(2):123–39. doi: 10.1177/106907279500300202

[50] Horwood S Anglim J Tooley G . Type d personality and the five-factor model: A facet-level analysis. Pers Individ Dif (2015) 83:50–4. doi: 10.1016/j.paid.2015.03.041

[51] Clarke D . Neuroticism: moderator or mediator in the relation between locus of control and depression? Pers Individ Dif (2004) 37(2):245–58. doi: 10.1016/j.paid.2003.08.015

[52] Mutlu T Balbag Z Cemrek F . The role of self-esteem, locus of control and big five personality traits in predicting hopelessness. Procedia-Social Behav Sci (2010) 9:1788–92. doi: 10.1016/j.sbspro.2010.12.401

[53] Costa PT Jr. McCrae RR . Four ways five factors are basic. Pers Individ Dif (1992) 13(6):653–65. doi: 10.1016/0191-8869(92)90236-I

[54] Hattrup K O’Connell MS Labrador JR . Incremental validity of locus of control after controlling for cognitive ability and conscientiousness. J Bus Psychol (2005) 19(4):461–81. doi: 10.1007/s10869-005-4519-1

[55] Helgeson VS . Implications of agency and communion for patient and spouse adjustment to a first coronary event. J Pers Soc. Psychol (1993) 64(5):807. doi: 10.1037/0022-3514.64.5.807 8505709

[56] Amanatullah ET Morris MW Curhan JR . Negotiators who give too much: unmitigated communion, relational anxieties, and economic costs in distributive and integrative bargaining. J Pers Soc Psychol (2008) 95(3):723. doi: 10.1037/a0012612 18729705

[57] Chapman BP Shah M Friedman B Drayer R Duberstein PR Lyness JM . Personality traits predict emergency department utilization over 3 years in older patients. Am J geriatr Psychiatry (2009) 17(6):526–35. doi: 10.1097/JGP.0b013e3181a2fbb1 PMC2745738 19461261

[58] Lippke S Pomp S Fleig L . Rehabilitants’ conscientiousness as a moderator of the intention–planning-behavior chain. Rehabil Psychol (2018) 63(3):460. doi: 10.1037/rep0000210 30113201

[59] Gois C Barbosa A Ferro A Santos AL Sousa F Akiskal H . The role of affective temperaments in metabolic control in patients with type 2 diabetes. J Affect Disord (2011) 134(1-3):52–8. doi: 10.1016/j.jad.2011.05.021 21641045

[60] Hazrati-Meimaneh Z Amini-Tehrani M Pourabbasi A Gharlipour Z Rahimi F Ranjbar-Shams P . The impact of personality traits on medication adherence and self-care in patients with type 2 diabetes mellitus: The moderating role of gender and age. J. psychosom Res (2020) 136:110178. doi: 10.1016/j.jpsychores.2020.110178 32623192

[61] Vileikyte L Rubin RR Leventhal H . Psychological aspects of diabetic neuropathic foot complications: an overview. Diabetes/metab Res Rev (2004) 20(S1):S13–S8. doi: 10.1002/dmrr.437 15150807

[62] Stephan Y Sutin AR Luchetti M Canada B Terracciano A . Personality and HbA1c: Findings from six samples. Psychoneuroendocrinology (2020) 120:104782. doi: 10.1016/j.psyneuen.2020.104782 32659693 PMC9837711

[63] Chapman BP Hampson S Clarkin J . Personality-informed interventions for healthy aging: conclusions from a national institute on aging work group. Dev Psychol (2014) 50(5):1426–41. doi: 10.1037/a0034135 PMC3940665 23978300

[64] Sutin AR Costa PT Jr. Chan W Milaneschi Y Eaton WW Zonderman AB . I Know not to, but I can’t help it: Weight gain and changes in impulsivity-related personality traits. Psychol Sci (2013) 24(7):1323–8. doi: 10.1177/0956797612469212 PMC3713084 23630223

