# Supplementary material for: Personality and weight management in adults with type 2 diabetes: A systematic review
Source: Front Clin Diabetes Healthc. 2022 Nov 11;3:1044005. doi: 10.3389/fcdhc.2022.1044005 (PMC10012143; doi:10.3389/fcdhc.2022.1044005)
Supplement: Supplementary file 2 [file Table_2.docx]

# Supplementary material 2 Subject heading (Emtree, MeSH, Thesaurus of Psychological Index Terms, CINAHL Headings, SPORTDiscus Subject Terms) and key term search

**Embase Complete: subject heading (Emtree) and key term search results**

#226**#225 AND [embase]/lim NOT ([embase]/lim AND [medline]/lim)**

#225**#223 AND #224**

#224**#220 OR #222**

#223**#219 OR #221**

#222**#205 OR #206 OR #207 OR #208 OR #209 OR #210 OR #211 OR #212 OR #213 OR #214 OR #215 OR #216 OR #217 OR #218**

#221**#181 OR #182 OR #183 OR #184 OR #185 OR #186 OR #187 OR #188 OR #189 OR #190 OR #191 OR #192 OR #193 OR #194 OR #195 OR #196 OR #197 OR #198 OR #199 OR #200 OR #201 OR #202 OR #203 OR #204**

#220**#176 OR #177 OR #178 OR #179 OR #180**

#219**#168 OR #169 OR #170 OR #171 OR #172 OR #173 OR #174 OR #175**

#218**hba1c:ab,ti**

#217**'hba 1c':ab,ti**

#216**hba1:ab,ti**

#215**'hba(1c)':ab,ti**

#214**hypoglyc*:ab,ti**

#213**hyperglyc*:ab,ti**

#212**insulin:ab,ti**

#211**niddm:ab,ti**

#210**iddm:ab,ti**

#209**t1dm:ab,ti**

#208**t1d:ab,ti**

#207**t2dm:ab,ti**

#206**t2d:ab,ti**

#205**diabet*:ab,ti**

#204**'dispositional theory':ab,ti**

#203**disposition:ab,ti**

#202**temperament:ab,ti**

#201**hexaco:ab,ti**

#200**'international personality item pool':ab,ti**

#199**ipip:ab,ti**

#198**'trait theory':ab,ti**

#197**'type d personality':ab,ti**

#196**'type c personality':ab,ti**

#195**'type b personality':ab,ti**

#194**'type a personality':ab,ti**

#193**'neo-pi':ab,ti**

#192**'neo pi':ab,ti**

#191**'five factor model':ab,ti**

#190**'big five':ab,ti**

#189**'big 5':ab,ti**

#188**extroversion:ab,ti**

#187**extraversion:ab,ti**

#186**agreeableness:ab,ti**

#185**'open to experience':ab,ti**

#184**openness:ab,ti**

#183**neurotic*:ab,ti**

#182**conscientious*:ab,ti**

#181**personalit*:ab,ti**

#180**'hypoglycemia'/de**

#179**'hyperglycemia'/de**

#178**'non insulin dependent diabetes'/de**

#177**'glycemic control'/de**

#176**'diabetes mellitus'/de**

#175**'personality test'/exp**

#174**'type d behavior'/de**

#173**'type b behavior'/de**

#172**'type a behavior'/de**

#171**'temperament'/de**

#170**'introversion'/de**

#169**'extraversion'/de**

#168**'personality'/de**

#167**#166 AND 'human'/de AND [embase]/lim NOT ([embase]/lim AND [medline]/lim) AND ('Article'/it OR 'Article in Press'/it OR 'Conference Abstract'/it OR 'Conference Paper'/it OR 'Conference Review'/it OR 'Review'/it OR 'Short Survey'/it)**

#166**#164 AND #165**

#165**#161 OR #163**

#164**#160 OR #162**

#163**#146 OR #147 OR #148 OR #149 OR #150 OR #151 OR #152 OR #153 OR #154 OR #155 OR #156 OR #157 OR #158 OR #159**

#162**#122 OR #123 OR #124 OR #125 OR #126 OR #127 OR #128 OR #129 OR #130 OR #131 OR #132 OR #133 OR #134 OR #135 OR #136 OR #137 OR #138 OR #139 OR #140 OR #141 OR #142 OR #143 OR #144 OR #145**

#161**#117 OR #118 OR #119 OR #120 OR #121**

#160**#109 OR #110 OR #111 OR #112 OR #113 OR #114 OR #115 OR #116**

#159**hba1c:ab,ti**

#158**'hba 1c':ab,ti**

#157**hba1:ab,ti**

#156**'hba(1c)':ab,ti**

#155**hypoglyc*:ab,ti**

#154**hyperglyc*:ab,ti**

#153**insulin:ab,ti**

#152**niddm:ab,ti**

#151**iddm:ab,ti**

#150**t1dm:ab,ti**

#149**t1d:ab,ti**

#148**t2dm:ab,ti**

#147**t2d:ab,ti**

#146**diabet*:ab,ti**

#145**'dispositional theory':ab,ti**

#144**disposition:ab,ti**

#143**temperament:ab,ti**

#142**hexaco:ab,ti**

#141**'international personality item pool':ab,ti**

#140**ipip:ab,ti**

#139**'trait theory':ab,ti**

#138**'type d personality':ab,ti**

#137**'type c personality':ab,ti**

#136**'type b personality':ab,ti**

#135**'type a personality':ab,ti**

#134**'neo-pi':ab,ti**

#133**'neo pi':ab,ti**

#132**'five factor model':ab,ti**

#131**'big five':ab,ti**

#130**'big 5':ab,ti**

#129**extroversion:ab,ti**

#128**extraversion:ab,ti**

#127**agreeableness:ab,ti**

#126**'open to experience':ab,ti**

#125**openness:ab,ti**

#124**neurotic*:ab,ti**

#123**conscientious*:ab,ti**

#122**personalit*:ab,ti**

#121**'hypoglycemia'/de**

#120**'hyperglycemia'/de**

#119**'non insulin dependent diabetes'/de**

#118**'glycemic control'/de**

#117**'diabetes mellitus'/de**

#116**'personality test'/exp**

#115**'type d behavior'/de**

#114**'type b behavior'/de**

#113**'type a behavior'/de**

#112**'temperament'/de**

#111**'introversion'/de**

#110**'extraversion'/de**

#109**'personality'/de**

#108**#107 AND ('clinical article'/de OR 'clinical trial'/de OR 'cohort analysis'/de OR 'comparative study'/de OR 'controlled clinical trial'/de OR 'controlled study'/de OR 'cross-sectional study'/de OR 'human'/de OR 'human experiment'/de OR 'longitudinal study'/de OR 'major clinical study'/de OR 'model'/de OR 'normal human'/de OR 'prospective study'/de OR 'questionnaire'/de OR 'randomized controlled trial'/de OR 'retrospective study'/de) AND ('Article'/it OR 'Article in Press'/it OR 'Conference Paper'/it) AND ([adult]/lim OR [aged]/lim OR [middle aged]/lim OR [very elderly]/lim OR [young adult]/lim)**

#107**#105 AND #106**

#106**#75 OR #84**

#105**#98 OR #104**

#104**#99 OR #100 OR #101 OR #102 OR #103**

#103**'hypoglycemia'/de**

#102**'hyperglycemia'/de**

#101**'non insulin dependent diabetes mellitus'/de**

#100**'glycemic control'/de**

#99**'diabetes mellitus'/de**

#98**#85 OR #86 OR #87 OR #88 OR #89 OR #90 OR #91 OR #92 OR #93 OR #94 OR #95 OR #96 OR #97**

#97**'hba1c':ab,ti**

#96**'hba1':ab,ti**

#95**'hba(1c)':ab,ti**

#94**'hypoglyc*':ab,ti**

#93**'hyperglyc*':ab,ti**

#92**'insulin':ab,ti**

#91**'niddm':ab,ti**

#90**'iddm':ab,ti**

#89**'t1dm':ab,ti**

#88**'t1d':ab,ti**

#87**'t2dm':ab,ti**

#86**'t2d':ab,ti**

#85**'diabet*':ab,ti**

#84**#76 OR #77 OR #78 OR #79 OR #80 OR #81 OR #82 OR #83**

#83**'personality test'/exp**

#82**'type d behavior'/de**

#81**'type b behavior'/de**

#80**'type a behavior'/de**

#79**'temperament'/de**

#78**'introversion'/de**

#77**'extraversion'/de**

#76**'personality'/de**

#75**#55 OR #56 OR #57 OR #58 OR #59 OR #60 OR #61 OR #62 OR #63 OR #64 OR #65 OR #66 OR #67 OR #68 OR #69 OR #70 OR #71 OR #72 OR #73 OR #74**

#74**'dispositional theory':ab,ti**

#73**'disposition':ab,ti**

#72**'temperament':ab,ti**

#71**'hexaco':ab,ti**

#70**'trait theory':ab,ti**

#69**'type d personality':ab,ti**

#68**'type c personality':ab,ti**

#67**'type b personality':ab,ti**

#66**'type a personality':ab,ti**

#65**'five factor model':ab,ti**

#64**'big five':ab,ti**

#63**'big 5':ab,ti**

#62**'extroversion':ab,ti**

#61**'extraversion':ab,ti**

#60**'agreeableness':ab,ti**

#59**'open to experience':ab,ti**

#58**'openness':ab,ti**

#57**'neurotic*':ab,ti**

#56**'conscientious*':ab,ti**

#55**'personalit*':ab,ti**

#54**#53 AND [adult]/lim**

#53**#31 AND #52**

#52**#37 OR #51**

#51**#38 OR #39 OR #40 OR #41 OR #42 OR #43 OR #44 OR #45 OR #46 OR #47 OR #48 OR #49 OR #50**

#50**'hba1c':ab,ti**

#49**'hba1':ab,ti**

#48**'hba(1c)':ab,ti**

#47**'hypoglyc*':ab,ti**

#46**'hyperglyc*':ab,ti**

#45**'insulin':ab,ti**

#44**'niddm':ab,ti**

#43**'iddm':ab,ti**

#42**'t1dm':ab,ti**

#41**'t1d':ab,ti**

#40**'t2dm':ab,ti**

#39**'t2d':ab,ti**

#38**'diabet*':ab,ti**

#37**#32 OR #33 OR #34 OR #35 OR #36**

#36**'hypoglycemia'/de**

#35**'hyperglycemia'/de**

#34**'non insulin dependent diabetes mellitus'/de**

#33**'glycemic control'/de**

#32**'diabetes mellitus'/de**

#31**#9 OR #30**

#30**#10 OR #11 OR #12 OR #13 OR #14 OR #15 OR #16 OR #17 OR #18 OR #19 OR #20 OR #21 OR #22 OR #23 OR #24 OR #25 OR #26 OR #27 OR #28 OR #29**

#29**'dispositional theory':ab,ti**

#28**'disposition':ab,ti**

#27**'temperament':ab,ti**

#26**'hexaco':ab,ti**

#25**'trait theory':ab,ti**

#24**'type d personality':ab,ti**

#23**'type c personality':ab,ti**

#22**'type b personality':ab,ti**

#21**'type a personality':ab,ti**

#20**'five factor model':ab,ti**

#19**'big five':ab,ti**

#18**'big 5':ab,ti**

#17**'extroversion':ab,ti**

#16**'extraversion':ab,ti**

#15**'agreeableness':ab,ti**

#14**'open to experience':ab,ti**

#13**'openness':ab,ti**

#12**'neurotic*':ab,ti**

#11**'conscientious*':ab,ti**

#10**'personalit*':ab,ti**

#9**#1 OR #2 OR #3 OR #4 OR #5 OR #6 OR #7 OR #8**

#8**'personality test'/exp**

#7**'type d behavior'/de**

#6**'type b behavior'/de**

#5**'type a behavior'/de**

#4**'temperament'/de**

#3**'introversion'/de**

#2**'extraversion'/de**

#1**'personality'/de**

**MEDLINE Complete: subject heading (MeSH) and key term search results**

**Personality subject headings:** Personality OR “Personality Development” OR “Personality Assessment” OR “Personality Inventory” OR “Personality Tests” OR “Type D Personality” OR “Type B Personality” OR Type A Personality” OR “Introversion (Psychology)” OR “Extraversion (Psychology)”

**Personality key terms:** Personalit* OR conscientious* OR neurotic* OR openness OR “open to experience” OR agreeableness OR extraversion OR extroversion OR “Big 5” OR “Big Five” OR “Five Factor Model” OR “NEO PI” OR “NEO-PI” OR “Type A Personality” OR “Type B Personality” OR “Type C Personality” OR “Type D Personality” OR “trait theory” OR IPIP OR “International Personality Item Pool” HEXACO OR temperament OR disposition OR “dispositional theory”

**Diabetes subject headings:** “Diabetes Mellitus” OR “Diabetes Mellitus, Type 2” OR Hyperglycemia OR Hypoglycemia

**Diabetes key terms:** Diabet* OR T2D OR T2DM OR T1D OR T1DM OR IDDM OR NIDDM OR insulin OR hyperglyc* OR hypoglyc* OR HbA(1c) OR HbA1 OR “HbA 1c” OR HbA1c

**PsychINFO: subject heading and key term search results**

**Personality subject headings:** Personality OR “Personality Measures” OR “Personality Differences” OR “Personality Traits” OR “Personality Theory” OR “Personality Correlates” OR “Five Factor Personality Model” OR “Individual Differences” OR “NEO Personality Inventory” OR “Nonprojective Personality Measures” (exploded) OR “Coronary Prone Behavior”

**Personality key terms:** Personalit* OR conscientious* OR neurotic* OR openness OR “open to experience” OR agreeableness OR extraversion OR extroversion OR “Big 5” OR “Big Five” OR “Five Factor Model” OR “NEO PI” OR “NEO-PI” OR “Type A Personality” OR “Type B Personality” OR “Type C Personality” OR “Type D Personality” OR “trait theory” OR IPIP OR “International Personality Item Pool” HEXACO OR temperament OR disposition OR “dispositional theory”

**Diabetes subject headings:** Diabetes OR “Diabetes Mellitus” OR “Type 2 Diabetes” OR “Blood Sugar”

**Diabetes key terms:** Diabet* OR T2D OR T2DM OR T1D OR T1DM OR IDDM OR NIDDM OR insulin OR hyperglyc* OR hypoglyc* OR HbA(1c) OR HbA1 OR “HbA 1c” OR HbA1c

**CINAHL Complete: subject heading (CINAHL Subject Headings) and key term search results**

**Personality subject headings:** Personality OR “Personality Development” OR “Personality Assessment” OR “Personality Tests”

**Personality key terms:** Personalit* OR conscientious* OR neurotic* OR openness OR “open to experience” OR agreeableness OR extraversion OR extroversion OR “Big 5” OR “Big Five” OR “Five Factor Model” OR “NEO PI” OR “NEO-PI” OR “Type A Personality” OR “Type B Personality” OR “Type C Personality” OR “Type D Personality” OR “trait theory” OR IPIP OR “International Personality Item Pool” HEXACO OR temperament OR disposition OR “dispositional theory”

**Diabetes subject headings:** “Diabetes Mellitus” OR “Diabetes Mellitus, Type 2” OR “Glycemic Control” OR Hyperglycemia OR Hypoglycemia

**Diabetes key terms:** Diabet* OR T2D OR T2DM OR T1D OR T1DM OR IDDM OR NIDDM OR insulin OR hyperglyc* OR hypoglyc* OR HbA(1c) OR HbA1 OR “HbA 1c” OR HbA1c

**SPORTDiscus with Full Text: subject heading and key term search results**

**Personality subject headings:** Personality OR Character OR Conscientiousness OR “Resilience (Personality Trait)” OR “Type A Behavior”

**Personality key terms:** Personalit* OR conscientious* OR neurotic* OR openness OR “open to experience” OR agreeableness OR extraversion OR extroversion OR “Big 5” OR “Big Five” OR “Five Factor Model” OR “NEO PI” OR “NEO-PI” OR “Type A Personality” OR “Type B Personality” OR “Type C Personality” OR “Type D Personality” OR “trait theory” OR IPIP OR “International Personality Item Pool” HEXACO OR temperament OR disposition OR “dispositional theory”

**Diabetes subject headings:** Diabetes OR “Glycemic Control” OR “Non-Insulin-Dependent Diabetes” OR Hyperglycemia OR Hypoglycemia

**Diabetes key terms:** Diabet* OR T2D OR T2DM OR T1D OR T1DM OR IDDM OR NIDDM OR insulin OR hyperglyc* OR hypoglyc* OR HbA(1c) OR HbA1 OR “HbA 1c” OR HbA1c
